# 693. Clinical and Economic Outcomes Associated with Fidaxomicin in Comparison to Vancomycin, Metronidazole, and FMT: a Systematic Literature Review

**DOI:** 10.1093/ofid/ofad500.755

**Published:** 2023-11-27

**Authors:** Qinghua Li, Engels N Obi, Anne Marciniak, Rebecca Newman

**Affiliations:** Merck & Co., Inc.,, Rahway, New Jersey; Merck & Co., Inc, BASKING RIDGE, New Jersey; Adelphi Values PROVE, Bollington, England, United Kingdom; Adelphi Values PROVE, Bollington, England, United Kingdom

## Abstract

**Background:**

*Clostridioides difficile* causes *Clostridioides difficile* infection (CDI), of which there are estimated half a million cases in the United States (US) each year. Fidaxomicin, vancomycin, and metronidazole are commonly used for the treatment of CDI, and fidaxomicin is recommended by clinical guidelines as the preferred treatment for patients with initial and recurrent CDI.

The aim of this systematic literature review was to explore the clinical and economic outcomes associated with the use of fidaxomicin with or without comparison to vancomycin, metronidazole, or fecal microbiota transplantation (FMT).

**Methods:**

The Embase, Medline, EconLit, and Evidence Based Medicine (EBM) Reviews databases were searched from 1^st^ January 2012 to 6^th^ December 2022. Identified publications were assessed and extracted by two independent reviewers.

**Results:**

Overall, 82 publications were included. In studies with ≥50 patients, ≥90 days post-treatment, clinical cure rate was 68–97% for fidaxomicin (N=12) and 66–92% for vancomycin (N=5), recurrence rate was 0–36% for fidaxomicin (N=19) and 17–29% for vancomycin (N=7), and all-cause mortality rate was 0–23% with fidaxomicin (N=4) and 4–39% with vancomycin (N=3). No relevant clinical outcomes were identified with metronidazole or FMT. There were limited healthcare resource use data. In studies with ≥50 patients (N=3), median hospital stay with fidaxomicin was 5.6–19.0 days.

Of 20 publications reporting cost-effectiveness, fidaxomicin was cost-effective compared to vancomycin in twelve, metronidazole in four, and FMT in one, as first-line treatment for non-severe CDI and in populations at high risk of recurrence. Six of 20 studies reported vancomycin or FMT as more cost-effective than fidaxomicin. Six of seven publications including patients aged ≥60 years, with cancer, renal impairment, or concomitant antibiotics found fidaxomicin cost-effective compared to vancomycin. Six publications stated that the high acquisition cost of fidaxomicin was offset by reduced recurrence and associated HCRU.

**Conclusion:**

Fidaxomicin was clinically effective compared to vancomycin. Fidaxomicin is often reported as cost-effective compared to vancomycin, consistently within high-risk subpopulations.

**Disclosures:**

**Qinghua Li, PhD**, Merck & Co Inc: Employee **Engels N. Obi, PhD**, Merck & Co Inc: Employee **Anne Marciniak, MD, PhD, MPhil, MSc**, Abbvie: Stocks/Bonds|Amgen: Stocks/Bonds|Pfizer: Stocks/Bonds

